# Expert consensus on neurodevelopmental outcomes in pregnancy pharmacovigilance studies

**DOI:** 10.3389/fphar.2023.1094698

**Published:** 2023-06-01

**Authors:** R. L. Bromley, M. Bickle Graz, M. Bluett-Duncan, C. Chambers, P. Damkier, K. Dietrich, H. Dolk, K. Grant, S. Mattson, K. J. Meador, H. Nordeng, T. F. Oberlander, A. Ornoy, A. Revet, J. Richardson, J. Rovet, L. Schuler-Faccini, E. Smearman, V. Simms, C. Vorhees, K. Wide, A. Wood, L. Yates, E. Ystrom, T. A. Supraja, J. Adams

**Affiliations:** ^1^ Division of Neuroscience, Faculty of Medicine, School of Biological Sciences, Biology and Health, University of Manchester, Manchester, United Kingdom; ^2^ Royal Manchester Children’s Hospital, Manchester Academic Sciences Park, Manchester, United Kingdom; ^3^ Neonatology, Department Woman-Mother-Child, Lausanne University Hospital, Lausanne, Switzerland.; ^4^ Division of Environmental Science and Health, Department of Pediatrics, University of California San Diego School of Medicine, San Diego, CA, United States; ^5^ Department of Clinical Pharmacology, Odense University Hospital, Denmark and Department of Clinical Research, University of Southern Denmark, Odense, Denmark; ^6^ Department of Environmental and Public Health Sciences, Division of Epidemiology, University of Cincinnati College of Medicine, Cincinnati, OH, United States; ^7^ Maternal, Fetal and Infant Research Unit, Faculty of Life and Health Sciences, Ulster University, Coleraine, United Kingdom; ^8^ Department of Environmental and Occupational Health Sciences, School of Public Health, University of Washington, Seattle, WA, United States; ^9^ Center for Behavioral Teratology, San Diego State University, San Diego, CA, United States; ^10^ Department of Neurology and Neurological Sciences, Stanford University School of Medicine, Palo Alto, CA, United States; ^11^ Pharmacoepidemiology and Drug Safety Research Group, Department of Pharmacy, University of Oslo, Oslo, Norway; ^12^ Department of Child Health and Development, Norwegian Institute of Public Health, Oslo, Norway; ^13^ Department Pediatrics and School of Population and Public Health, University of British Columbia, Vancouver, BC, Canada; ^14^ Hebrew University Hadassah Medical School, Adelson School of Medicine, Ariel University, Jerusalem, Israel; ^15^ INSERM University of Toulouse 3, Toulouse, France; ^16^ Department of Child and Adolescent Psychiatry, Toulouse University Hospital, Toulouse, France; ^17^ UK Teratology Information Service, Newcastle, United Kingdom; ^18^ Department of Psychology, University of Toronto, Toronto, ON, Canada; ^19^ The Hospital for Sick Children, Toronto, ON, Canada; ^20^ Genetics Department, Hospital de Clinicas de Porto Alegre, Universidade Federal Do Rio Grande Do Sul, Porto Alegre, Brazil; ^21^ Department of Surgery, Emory University, Atlanta, GA, United States; ^22^ School of Psychology, Faculty of Life and Health Sciences, Ulster University, Coleraine, United Kingdom; ^23^ Department of Pediatrics, Division of Neurology, University of Cincinnati College of Medicine and Cincinnati Children’s Hospital Medical Center, Cincinnati, OH, United States; ^24^ Department of Pediatrics, CLINTEC, Karolinska Institutet, Karolinska University Hospital, Stockholm, Sweden; ^25^ Clinical Sciences, Murdoch Children’s Research Institute, Melbourne, VIC, Australia; ^26^ Northern Genetics Service, Newcastle Upon Tyne Hospitals NHS Foundation Trust, Newcastle, United Kingdom; ^27^ KRISP, University of KwaZulu-Natal, Durban, South Africa; ^28^ Department of Mental Disorders, Norwegian Institute of Public Health, Oslo, Norway; ^29^ PROMENTA Research Center, Department of Psychology, University of Oslo, Oslo, Norway; ^30^ National Institute of Mental Health and Neurosciences NIMHANS, Bangalore, Karnataka, India; ^31^ Department of Psychology, University of Massachusetts Boston, Boston, MA, United States

**Keywords:** pharmacovigilance, medicines, pregnancy, neurodevelopment, neurobehavior, teratogens, in utero exposure

## Abstract

**Background:** Exposure *in utero* to certain medications can disrupt processes of fetal development, including brain development, leading to a continuum of neurodevelopmental difficulties. Recognizing the deficiency of neurodevelopmental investigations within pregnancy pharmacovigilance, an international Neurodevelopmental Expert Working Group was convened to achieve consensus regarding the core neurodevelopmental outcomes, optimization of methodological approaches and barriers to conducting pregnancy pharmacovigilance studies with neurodevelopmental outcomes.

**Methods:** A modified Delphi study was undertaken based on stakeholder and expert input. Stakeholders (patient, pharmaceutical, academic and regulatory) were invited to define topics, pertaining to neurodevelopmental investigations in medication-exposed pregnancies. Experts were identified for their experience regarding neurodevelopmental outcomes following medicinal, substances of misuse or environmental exposures *in utero*. Two questionnaire rounds and a virtual discussion meeting were used to explore expert opinion on the topics identified by the stakeholders.

**Results:** Twenty-five experts, from 13 countries and professionally diverse backgrounds took part in the development of 11 recommendations. The recommendations focus on the importance of neurodevelopment as a core feature of pregnancy pharmacovigilance, the timing of study initiation and a core set of distinct but interrelated neurodevelopmental skills or diagnoses which require investigation. Studies should start in infancy with an extended period of investigation into adolescence, with more frequent sampling during rapid periods of development. Additionally, recommendations are made regarding optimal approach to neurodevelopmental outcome measurement, comparator groups, exposure factors, a core set of confounding and mediating variables, attrition, reporting of results and the required improvements in funding for potential later emerging effects. Different study designs will be required depending on the specific neurodevelopmental outcome type under investigation and whether the medicine in question is newly approved or already in widespread use.

**Conclusion:** An improved focus on neurodevelopmental outcomes is required within pregnancy pharmacovigilance. These expert recommendations should be met across a complementary set of studies which converge to form a comprehensive set of evidence regarding neurodevelopmental outcomes in pregnancy pharmacovigilance.

## 1 Introduction

Exposure *in utero* to certain medications, chemicals and maternal diseases can disrupt processes of fetal development, leading to a continuum of outcomes, from those immediately evident such as embryo loss or major physical malformation, through to functional deficits (Vorhees and Riley, 1986). Fetal brain development *in utero*, although often overlooked, is also susceptible to the effects of a teratogenic exposure and can range from observable structural alterations through to functional difficulties (e.g., intellectual functioning) with no associated macroscopically visible brain abnormalities (Vorhees and Riley, 1986; Rice and Barone, 2000; Rodier, 2004; Grandjean and Landrigan, 2006; Adams, 2010). Perturbed development of the neuronal architecture can lead to a myriad of childhood neurodevelopmental difficulties including delays in early language and motor skill acquisition, lower IQ, poorer educational outcomes, attention deficit and hyperactivity disorder (ADHD) and autism spectrum disorders (ASD) (Meredith et al., 2015). Depending on the nature and gestational timing of the exposure, the exposure dose, duration and individual materno-fetal susceptibility factors (Adams et al., 2000; Rice and Barone, 2000), the neurodevelopmental difficulties can range from mild through to substantial and life impacting (Jacobson and Jacobson, 1996; Brent, 2004; Streissguth et al., 2004; Amler et al., 2006).

The term neurodevelopment refers to an independent but interlinked set of brain functions that evolve in a relatively predictable developmental pattern including intelligence, language, memory, attention, executive functions, motor, social, behavioral skills and includes clusters of symptoms which form specific clinical disorders (e.g., ASD, ADHD). Development is rapid in the first few years, but the process of skill acquisition and maturity continues into early adulthood (Rice and Barone, 2000; Arain et al., 2013). Disruptions to neurodevelopmental functioning can have lifelong implications and be costly for the individual, the family and society in terms of the support required. The average lifetime cost of intellectual disability (ID) or ASD is estimated to be around 1 million US dollars (Centers for Disease Control and Prevention, 2004; Buescher et al., 2014; Arora et al., 2020), and for ADHD around 300,000 US Dollars (Ornoy and Spivak, 2019), but varies by country.

Historically, pregnancy pharmacovigilance (PregPV) initiatives have not prioritized, or even included neurodevelopmental outcomes (Charlton and de Vries, 2016; Roque Pereira et al., 2022). However, recently regulators worldwide have become increasingly aware that the reproductive safety of medicines cannot be assured without knowledge of long-term neurodevelopmental outcomes as evidenced by the evolution in our knowledge of sodium valproate (Medicines and Healthcare Regulation Authority, 2021). Whilst pharmacovigilance guidelines have been updated (e.g., European Medicines Agency GVP III, U.S. Food and Drug Administration Postapproval Pregnancy Safety Studies Guidance for Industry) (European Medicines Agency, 2019; U.S. Food and Drug Administration, 2019), and include longer term neurodevelopmental outcome investigation, there is no specific guidance regarding the types of neurodevelopmental outcomes which are considered central to investigations nor guidance regarding the timing and nature of the investigations required in PregPV investigations.

The ConcePTION Project (https://www.imi-conception.eu/) is a collaboration between academia, industry and regulators that strives to improve PregPV. As part of its work, it aims to achieve improved PregPV including neurodevelopmental outcome investigations. Here we report the outcome of an Expert Consensus Delphi Study regarding neurodevelopmental research in the context of PregPV. The aim of this process was to develop expert guidance on key aspects of neurodevelopmental investigations including their importance, timing and optimal collection of data in PregPV studies, through multidisciplinary expert consultation and consensus.

## 2 Procedure

A Delphi study was undertaken to develop expert consensus regarding methodological aspects of PregPV investigations into neurodevelopmental outcomes. Stakeholders generated core themes which were then taken through three rounds of expert consultation to reach a consensus opinion. Each of these stages are described in more detail below.

### 2.1 Identification of core themes

Through the ConcePTION consortium, Stakeholders were approached via email and were invited to highlight key topics regarding neurodevelopmental outcomes in PregPV initiatives that, in their opinion, required expert consensus. Thirteen Stakeholders, including representatives from medicine regulators (n = 1), teratology information services (n = 3), pregnancy registers (n = 2), pharmaceutical companies (n = 2), patient groups (n = 3) and pharmacovigilance researchers (n = 2), provided their views on areas needing guidance from neurodevelopmental experts.

Topics identified by the Stakeholders as requiring expert consensus or guidance mapped on to the areas of 1. importance and timing of investigation, 2. core outcomes, 3. optimal methodologies, 4. age of children at investigation and, 5. when is evidence conclusive (Supplementary Table S1). An additional topic regarding the barriers to investigating neurodevelopmental outcomes was added to the list presented to the Neurodevelopmental Expert Working Group (NEWG).

### 2.2 Identification of experts

Input from a diverse group of researchers and clinicians with experience in neurodevelopmental outcomes following medicinal, substances of misuse or environmental exposures *in utero* was sought*.* Individuals with expertise across different research techniques and professional backgrounds were identified via literature search and membership lists of relevant groups (i.e., European Network of Teratology Information Services (ENTIS), European Network of Centres for Pharmacoepidemiology and Pharmacovigilance (ENCePP), and the Developmental Neurotoxicology Society (DNTS). Our aim was to recruit 20–25 experts from a wide range of PregPV, teratology or developmental toxicology backgrounds to provide an optimal group size for a healthcare Delphi study (Akins et al., 2005). Attempts were made to limit attrition by completing the process over a short time (10 weeks).

**FIGURE 1:**
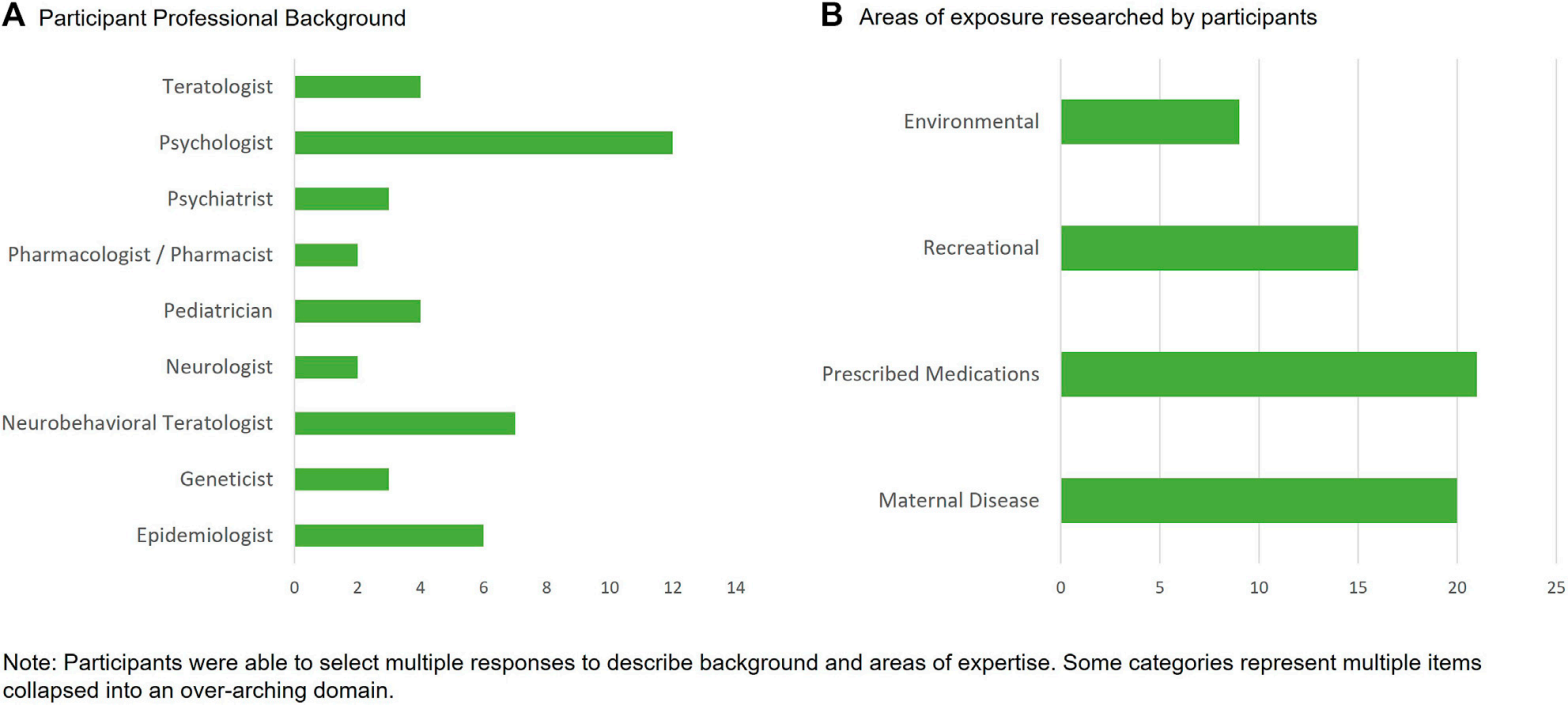
Professional background and expertise of the neurodevelopmental expert working group.

In total, 25 experts responded positively from 32 invitations (78%). The assembled group was diverse in their professional backgrounds and in the exposures they researched (Figure 1). The mean years of experience in researching neurodevelopmental outcomes was 22 years and ranged from 4 years to 44 years. Experts worked in 13 different countries and reported experience across a range of study design types including those involving primary data collection (n = 20), those with secondary use of routine health or education data (n = 13) and with preclinical study experience (n = 4).

### 2.3 Development of questions

A Delphi study approach (Jorm, 2015; Trevelyan and Robinson, 2015) was employed. Based on the four themes identified by the Stakeholders (Supplemental Table S1), plus the theme of barriers to neurodevelopmental research, a series of 14 open-ended questions was developed by a core group (authors RB, MBD, JA, LY, TC, PD) (Supplemental Table S2) and formed Round 1 of the process. Open questions included *‘from your experience and the literature which aspects of neurodevelopment can be altered by prenatal exposures?’* and *‘what areas of neurodevelopment would you consider important enough to have needed investigation before any conclusion about risk or safety can be made?’.* During round 1, experts were also able to propose new themes/questions.

The Round 1 free text/open responses and suggestions were collated, and a thematic analysis approach used to create Round 2 closed questions/statements to allow for aggregation of the responses to obtain an objective consensus (Supplementary Table S3). Each Round 2 item was preceded by a brief narrative summarizing the responses given in Round 1. Respondents were typically asked to rate their response on a 5-point Likert scale, ranging from 1 (Strongly Disagree) to 5 (Strongly Agree). Several ‘multiple’ option questions were also used to allow respondents to select all answers which may apply, and free text commenting was also available for each question to add greater depth to responses. Expert responses to the first two rounds were anonymous and each question included an option to opt out of providing a response due to a lack of specific expertise.

### 2.4 Virtual meeting

An ‘in person’ virtual meeting (Round 3), chaired by an independent representative from the ConcePTION Project, was held and recorded to allow participation by two experts who were unavailable for the live meeting. During the meeting a structured review of the questions where there was clear consensus was presented. Where the first two rounds had failed to provide consensus or where there were high levels of variance in voting, discussions on these topics were held and a final round of voting was conducted.

### 2.5 Consensus generation

Responses from Rounds 2 and 3 were collated and median Likert-Scale scores and percentages of agreement were produced (Murphy et al., 1998). Initially, a median Likert Scale score above 4 or 80% of responses indicating that they ‘agreed’ or ‘strongly agreed’ was accepted as a threshold of consensus within the group. However, by Round 3 almost all questions had responses that met the median 4 criteria and therefore the 80% criterion was deemed to be more sensitive for determining consensus.

## 3 Results

Fourteen questions proposed in Round 1 led to 11 questions relating to neurodevelopmental outcomes in the context of PregPV studies which were further considered by the NEWG in Round 2 (Supplementary Table S3). Five topics that had either not reached consensus or had substantial variance in voting were discussed in Round 3 (prioritization of medications to be investigated, core/central neurodevelopmental domains, aspects of optimal investigation, confounder and mediators and comparator groups). The outcome of this process was 11 recommendations (Supplementary Table S4). Each Recommendation is discussed below, along with supporting evidence or examples from the literature. Statements were included when the panel reached consensus, which was defined as = />80% of experts ‘agreed’ or ‘strongly agreed’. Where consensus was not reached, or it was noteworthy, percentages of agreement are provided.

### 3.1 Neurodevelopmental outcomes in pregnancy pharmacovigilance

**Table:** 

Recommendation 1
Neurodevelopmental outcomes should be integral to pregnancy pharmacovigilance

The NEWG highlighted that the scientific evidence demonstrates unequivocally that the developing brain is highly susceptible to several environmental (e.g., lead) and recreational (e.g., alcohol) exposures, as well as certain medications (e.g., isotretinoin, valproate, phenobarbital) (Thompson et al., 2009). Neurodevelopmental deficits associated with such exposures have been shown to have substantial life changing and lifelong implications, often with no corrective treatments available (although early intervention may ameliorate certain outcomes). There are therefore high social and emotional impacts for affected families, as well as the considerable financial implications with social care and healthcare related costs.

In addition to the severity of the impact, the NEWG noted that neurodevelopmental deficits can be seen in the absence of, or more frequently than, structural anomalies (Vorhees and Riley, 1986), and these deficits can present at a lower dose threshold than that required for structural anomalies (Vorhees and Riley, 1986). This has been elucidated clinically for a range of exposures including alcohol (Riley and McGee, 2005), environmental exposures (Grandjean and Landrigan, 2006) and medications such as isotretinoin and valproate (Adams and Lammer, 1993; Meador et al., 2013). Therefore, based on the strength of scientific evidence of potential harm, and the frequency and significance of the adverse neurodevelopmental outcomes, the NEWG recommends that investigations into the potential effects of *in utero* exposures on the developing brain should be a central feature of PregPV.

### 3.2 When should neurodevelopmental investigation or surveillance be implemented?

**Table:** 

Recommendation 2
Neurodevelopmental investigations should automatically be part of the PregPV initiatives for a medication where one or more apply: i) It is mechanistically plausible for the medication to be associated with an increased risk or it belongs to a class of medications where effects have been observed (e.g., central nervous system acting medications) ii) There is evidence (preclinical or human data) of a higher risk to physical development (e.g., structural anomalies) iii) There is evidence (preclinical or human data) of a higher risk to brain development iv) The medication is likely to be widely used among women of childbearing potential

Stakeholder input requested guidance regarding when neurodevelopmental investigations should be initiated. Seventy-six percent of NEWG members felt that investigations into the potential for human neurodevelopmental risks should be included as a routine or mandated investigation for all medications following regulatory approval. However, points of concern relating to the logistic and financial feasibility of this were raised by NEWG members who did not support routine investigations for all medications.

Further discussion on this topic led to a pragmatic approach of identifying the medications for which risk of altered neurodevelopmental outcomes was considered greatest, prioritizing these for investigation. The agreed upon criteria fell into three categories, which are displayed in Table 1, along with NEWG voting patterns. The importance of preclinical work (e.g., animal or human cell models) was highlighted in group voting and in Round 3 discussions. An impact on the developing nervous system in preclinical investigations must lead to human investigations and the group noted in discussions previous alignment between preclinical and human studies for both environmental exposures and certain medications (Rice and Barone, 2000; Rodier, 2004; Adams, 2010).

**TABLE 1:** Key factors for selecting medication for routine human research.

Factor^a^	Strongly disagree (%)	Disagree (%)	Nether agree nor disagree (%)	Agree (%)	Strongly agree (%)
Central nervous system acting medications	4.0	0.0	0.0	16.0	80.0
Documented physical impact	0.0	0.0	0.0	16.0	84.0
Documented animal risk	4.2	4.2	4.2	29.2	58.3
Widely used in relevant population	0.0	4.0	8.0	44.0	48.0
Plausible mechanism/class	0.0	4.2	0.0	16.7	79.2
Published evidence of risk	0.0	4.0	8.0	28.0	60.0
Regulator/Industry signal	0.0	0.0	8.7	43.5%	47.8

^a^ Factors derived via Round 1 free text reponses. Number of participants voting for each factor may vary due to participants opting out of certain items due to lack of expertise. All items voted on as part of Round 2 questions.

Where one or more of these criteria are met regulators should to a larger extent make neurodevelopmental safety studies mandatory, and where there is an intersection of two or more of these criteria, investigations are required as a priority and should be sufficiently detailed to support early identification of a signal and additionally a comprehensive evidence base thereafter. For example, central nervous system acting medications were specifically highlighted by the NEWG as medications which should be prioritized for routine human (e.g., clinical or epidemiological) investigation at point of market authorization/approval (96% agreement), due to their mechanistic routes of action and that several potent neurodevelopmental teratogens have been found within this class of medications.

More challenging, is that these criteria should also be applied, to already approved and used medicines. Most currently approved medicines have little to no data pertaining to neurodevelopmental outcomes, even where there are known structural or physical risks, or for medication classes with known risk of neurodevelopmental impact, such as the antiseizure medications (Knight et al., 2021). Stakeholders will need to work collaboratively to devise an approach to already approved medications, without comprehensive neurodevelopmental outcome data, using these recommendations.

### 3.3 Core neurodevelopmental outcomes for pregnancy pharmacovigilance

**Table:** 

Recommendation 3
An evidence base must include assessment of these expert agreed neurodevelopmental functions. These include cognitive, motor, behavior and emotional functioning as well as clinical disorders and educational outcomes

Neurodevelopment is an encompassing term for a range of different, but interlinked, functions of the brain. Developmental trajectories across a range of neurodevelopmental outcomes were identified by the NEWG as being susceptible to alterations via exposure to teratogens during pregnancy. The group strongly concluded (92% agreement) that no single neurodevelopmental outcome was able to summarize functioning across other neurodevelopmental areas. Whilst there is a degree of co-occurrence across certain neurodevelopmental outcomes there is also independence, which was recognized. For example, children with intellectual difficulties have a greater chance of also having a diagnosis of an autistic spectrum condition but, these conditions may also occur separately in the context of typical functioning in other neurodevelopmental domains. Further, different neural networks and the functions they support may vary in their sensitivity, or timing of their sensitivity, to *in utero* exposure to different agents, leading to specific neurodevelopmental profiles (Jacobson and Jacobson, 1996). This point is highlighted by the cognitive profiles of children exposed to isotretinoin and valproate, where isotretinoin is associated with relatively spared verbal functioning (Adams and Lammer, 1993), whilst this is a particular area of difficulty within the valproate-associated phenotype (Bromley et al., 2019). Thus, measurement within a single prioritized area of functioning cannot provide information that generalizes as an assessment of the integrity of functioning in other areas. Our recommendation is that measurement across multiple neurodevelopmental domains must be employed to avoid erroneous conclusions based on insufficient information.

Two distinct sub-types of neurodevelopmental outcomes emerged from the three rounds and are conceptualized as functional skills or clinical disorders (Forns et al., 2012) and are displayed in Table 2 along with levels of agreement. Functional skills include abilities, capacities, and knowledge acquired during the maturation of the brain (Forns et al., 2012) and are often only identified through specialized clinical evaluations. Functional skills such as cognition, including intellectual abilities, language, attention and memory functioning, received the highest support for investigation (Table 2), due to their observed sensitivity to several teratogenic exposures; both from medications and other exposure types (Adams and Lammer, 1993; Mattson et al., 2001; Julvez et al., 2007; Freire et al., 2010; Bromley et al., 2014; Munoz-Rocha et al., 2018). Clinical disorders (e.g., autistic spectrum disorder, attention deficit hyperactivity disorder) refer to the presence of a discrete set of predefined symptoms, which may sit across a range of different functional skills and likely represent the most severe end of a broad symptom continuum. High ratings of support (87.5%) were also given for the investigation of clinical neurodevelopmental disorder diagnoses by the NEWG (Table 2), which may sit within a broader set of symptoms associated with the exposure. As well as primary or direct impacts on neurodevelopmental outcomes, it was also noted that there are possible secondary or indirect impacts on everyday life, and these too should be considered for investigation. For example, a medication capable of inducing alterations in the development of the neuronal architecture may lead to poorer academic outcomes through reduced cognitive (e.g., intellectual, memory, executive) functioning or capacity.

**TABLE 2:** Neurodevelopmental functioning domains and clinical disorders known to be vulnerable to disruption through neurotoxin exposure and domains voted to be part of the core domain set.

Domain	Final level of agreement^a^ ^,^ ^b^	Domain	Level of agreement^a^ ^,^ ^b^ (%)
Consensus for core domain		Vulnerable to disruption through neurotoxin exposure	
Cognitive Functioning	100.0%	General cognitive ability^c^	100
		Learning rate	95.8
		Executive Function	95.7
		Attention	95.8
		Processing speed	95.7
		Expressive Language	95.2
		Memory	91.7
		Receptive Language	90.9
		Visuo-Spatial skills	90.5
Motor Skills	91.7%	Fine Motor	87.0
		Developmental Milestones	83.3
		Gross Motor	83.3
		Reflexes	63.1
Behaviour	94.1% (78.3%^b^)	Behaviour Problems	95.8
Emotions & Mood	88.2% (71.4%^b^)	Emotional Regulation	86.4
		Anxiety	72.3
		Depression	72.3
Proxy Outcomes	94.7% (57.9%^b^)	Exams	90.0
		Special Educational Needs requirement	90.9
		Occupation	66.6
Neurodevelopmental Clinical Disorders	87.5%	Clinical Disorders	95.8
Adaptive Functioning	77.8% (71.4%^b^)	Adaptive Behaviour	95.7
Social Functioning	77.8% (71.4%^b^)	Social Skills	87.5

^a^ Combined % of participants responding “Agree” or “Strongly Agree”.

^b^ Round 2 % agreement before discussion and second voting as part of Round 3 online meeting where itesm were discussed.

^c^ for example developmental quotient or intelligence quotient.

Although this NEWG did not explicitly produce age-related recommendations, the list of core domains will require tailoring to the age of the cohort. A similar consensus initiative focusing on outcomes in children from vaccinated cohorts, concluded that under the age of 5 years motor (fine and gross), language (receptive, expressive) and cognitive skills should be considered as key emerging domains which require comprehensive investigation (Villagomez et al., 2019). However, in school-aged children a wider range of neurodevelopmental processes will have emerged and therefore the range of neurodevelopmental outcomes should reflect this maturation (Dietrich et al., 2005).

This core outcome set for neurodevelopmental investigations should be viewed as the minimum evidence base required to reliably assess neurodevelopmental risk or relative safety. At an individual study level, it may not always be possible to include measurement of all these neurodevelopmental outcomes, but initiation of measurement of a broad range of domains should be considered for medication exposures, as in recommendations for other exposures (Dietrich et al., 2005; Grandjean and Landrigan, 2006). As an overall evidence base however, evidence should be obtained for all recommended domains before investigations cease and conclusions are made.

### 3.4 Optimizing methodological approaches to neurodevelopmental outcomes in pregnancy pharmacovigilance

First, as an open question and secondly as a series of statements, the NEWG identified methodological approaches required for the optimum collection of neurodevelopmental data for PregPV studies. The NEWG recognized that optimal methodological approaches would vary across domains of neurodevelopmental functioning, age at measurement and by methodological approach. However, the NEWG identified principles relating to the different aspects of neurodevelopmental study designs, which should be optimized where possible.

#### 3.4.1 The measurement of neurodevelopmental outcomes

**Table:** 

Recommendation 4
Neurodevelopmental outcomes are diverse and require different measurement approaches that are sensitive to deviations over time. Use of a standardized set of direct assessments, by trained assessors blinded to exposure status for the whole cohort receives the highest recommendation for measurement sensitivity

The optimal approach to the measurement of neurodevelopmental functions and clinical disorders may vary by domain, diagnosis, child age and study design. However, concepts were identified which were regarded as important by the NEWG to provide higher quality measurement, with a reduced chance of measurement error. Features of optimal measurement of neurodevelopmental outcomes (receiving = />80% agreement) included:

• Direct assessment of children by expert/highly trained assessors,
• A standardized assessment of all exposed and comparator children,
• Use of standardized and validated assessment measures (for example a psychometric instruments or diagnostic criteria),
• Blinding or masking of the assessors to the exposure group/history.

Assessments conducted blinded to the exposure status of the child reduce the potential influence of rater bias, although it remains underused on PregPV studies (Hjorth et al., 2019). Conducting the same assessment across all included study participants improves measurement sensitivity, and the use of a measure with standardized administration procedures reduces measurement variation and error. Utilizing highly trained assessors was rated as important to overcome the challenges of engaging children in an assessment and for improving the quality of the data (Dietrich et al., 2005; Villagomez et al., 2019).

There was discussion in Round 3 around the utilization of parent ratings on standardized questionnaires. The role of parent-completed screening questionnaire has been subject to some controversy with regards to whether they represent conclusive level evidence (Damkier et al., 2015). NEWG discussions recognized that parents are not blinded to exposure history, may be anxious about their child’s outcome, and may interpret the standardized questions differently and that these lead to potential biases and interrater variances. However, it was also considered that parent completed assessment via questionnaires were important for certain outcomes (e.g., social skills, adaptive behavior). For example, social skills are unlikely to be observed optimally in the unfamiliar environment of a clinic or research room, particularly given that assessments usually involve the child and one adult rather than a group of peers. Likewise, obtaining valid young infant assessments can be challenging and supplementing with parent ratings can improve the ecological validity of the data. Therefore, although parent-completed questionnaires alone were not accepted by the NEWG to provide *definitive data* there was recognition that parent ratings were valuable for certain outcomes (e.g., infant development, adaptive behavior, social skills) and for screening activities, particularly in large cohorts. However, results arising from initial screening activities using parent-completed questionnaires in isolation would warrant complimentary blinded, objective investigations.

The secondary use of clinical or educational data collected for administrative or insurance purposes often used in epidemiological PregPV research was viewed with sensitivity concerns by the NEWG. In Round 2 only 25% of experts ‘agreed’ or ‘strongly agreed’ that secondary use of health record data would provide conclusive evidence in isolation. Concerns centered around the sensitivity and specificity of the data due to variability in diagnostic processes across clinicians, accuracy and frequency of reporting, a lack of active blinding of the clinician/assessor and that only a subset of the medication-exposed group is referred for and therefore receives a diagnostic assessment or review. It was also noted that such data would be biased towards those with more severe phenotypes, would not provide any data on sub-diagnostic level difficulties which may still have an impact on daily functioning and therefore represent important PregPV information. Following Round 3 discussions regarding the role of secondary use of routine healthcare data, there was consensus from the NEWG (88%) that utilization of such data sources were an important PregPV tool, particularly for detecting signals of poorer neurodevelopmental outcomes or for when larger populations are required (e.g., for rare diagnoses/outcomes). Where an elevated risk of a clinical neurodevelopmental disorder is identified, it should be considered that a continuum of symptoms sub-diagnosis threshold will exist and should be investigated.

No blanket recommendations are made regarding individual measurement approaches or specific tests or measurement tools due to the different testing requirements required through the lifespan, different tests and versions available internationally, and the possible differences in sensitivity of specific assessments/measures across different exposure groups. Research groups, pharmaceutical industry, and regulators are strongly encouraged to collaborate with professionals with academic or clinical expertise in the areas of neurodevelopmental functioning under investigation to develop a sensitive approach to measurement selection.

#### 3.4.2 Exposure variables

**Table:** 

Recommendation 5
Factors related to the medication exposure including the dose, gestational timing, duration, and route of administration during pregnancy should be key considerations and study designs should take heterogeneity and confounding by indication into account

Consistent with other areas of pharmacovigilance and previous positions on neurodevelopmental outcomes in PregPV investigations (Vorhees and Riley, 1986; Brent, 2004) the NEWG recommend several key factors relating to medicine exposure that require consideration in studies investigating neurodevelopmental outcomes. These include:

- Dose of the exposure.
- Gestational timing of the exposure (accounting for exposure half-life).
- Duration of the exposure.
- Medication changes (including stopping or starting and compliance).
- Route of administration.
- Mechanism of the drug or chemical exposure.
- Continued exposure via breastfeeding.

Inadequate consideration of these aspects of the exposure could introduce important misclassification biases, influence the findings (bias towards or away from the null), and potentially impact the generalizability of study results. The development of the brain is an evolving set of processes with different points of vulnerability at different gestational times and postnatally (Rice and Barone, 2000). Therefore, different timings and/or duration of the exposure may lead to different neurodevelopmental risks and severity of such alterations (Adams et al., 2000). Medications where there are high rates of short-term use or discontinuation are likely to be most difficult to investigate, due to the heterogeneity of exposure windows across participants. Dose of any chemical exposure is a key aspect of toxicological impact (Vorhees and Riley, 1986) (Wilson, 1972; Vorhees and Riley, 1986; Adams et al., 2000) and medications are no different. Exposure to valproate for example demonstrates a dose sensitive risk profile for both major congenital anomalies (Samren et al., 1999; Tomson et al., 2018) and neurodevelopmental outcomes (Christensen et al., 2013; Meador et al., 2013; Baker et al., 2015). Therefore, approaches should be undertaken to ensure that these important factors inherent in the medication exposure are included in all PregPV studies. Efforts have been made recently to develop core data elements for both PregPV primary and secondary data collection approaches (Damase-Michel and Wurst, 2021; Richardson et al., 2023).

The methods of collection of data regarding the medication exposure were rated by members of the NEWG. A combination of prospective self-report and medical records was most highly rated followed by biological samples that quantify exposure, prospective self-report alone, medical records alone and dispensing records alone.

#### 3.4.3 Developmental stage and intervals of neurodevelopmental investigations

**Table:** 

Recommendation 6
The investigation of neurodevelopmental outcomes should start in infancy with an extended period of investigation into adolescence. Investigation or sampling intervals should be more frequent during periods of rapid development or after signals of early developmental deviations

Assessment in infancy allows for early detection of deviations from the normal developmental trajectory. In the NEWG, there was unanimous recognition that neurodevelopmental impairments may emerge over time and therefore neurodevelopmental surveillance required a protracted period of investigation. The NEWG unanimously agreed that investigations into neurodevelopmental outcomes should include infant neurodevelopmental outcomes on the basis that major human neurobehavioral teratogens such as alcohol, valproate and isotretinoin have induced detectable delays in infant milestone attainment (Adams and Lammer, 1993; Bromley et al., 2010; Davies et al., 2011). Medication exposures with early deviations from typical developmental trajectories, or where there are signals from screening activities, should be further investigated with urgency using optimized investigations, as defined by the NEWG. Detecting early emerging patterns of risk are useful on an individual level (early referral for intervention), but also raise early warnings to regulators and clinicians, ultimately leading to fewer children being exposed to a higher risk medication than clinically necessary.

However, neurodevelopmental investigations must continue beyond the infant years to allow for the full delineation of the impact which may only become visible with maturation of different neurodevelopmental functions and/or improvement in the sensitivity of measurement. It is recognized that brain development extends into early adulthood (Rice and Barone, 2000; Arain et al., 2013) and with this consideration there was consensus that comprehensive investigations should include investigation up to at least 16 years of age (86% supported this length of investigational follow up). Measurement sensitivity increases with the child’s age (Jacobson and Jacobson, 1996) and terminating investigations while a developmental skill/function is early in its emergence may not provide reliable results as significant neurodevelopmental deficits may emerge in one or more areas later in the developmental trajectory (Anderson et al., 2011).

The age intervals of follow up for both primary data collection and secondary use of routine health data is recommended to vary by outcome type. There was consensus that intervals between investigations should be narrower in the preschool years, due to this being a period of rapid developmental change, which may allow for early identification of any divergent developmental trajectories. Assessment or analysis in the school aged years can have wider assessment intervals, but there was a recognition that when early developmental deviations were noted the follow up should be more frequent and other areas of neurodevelopmental outcome also investigated.

Finally, as per other similar consensus recommendations (Amler et al., 2006; Villagomez et al., 2019), studies employing longitudinal follow ups at different ages were unanimously supported here. Longitudinal designs allow for the timing and interval recommendations above to be implemented, with each additional assessment allowing for findings from earlier timepoints to be strengthened or challenged.

#### 3.4.4 Recruitment, cohort ascertainment and attrition

**Table:** 

Recommendation 7
Research can be improved by using a prospective design, representative samples of women using the medication and through limited attrition which is not systematic and imbalanced across groups

The NEWG noted that prospective ascertainment of cohorts and recording of data was critical to optimize and reduce bias in neurodevelopmental outcome research. Establishing cohorts of children with a target medication exposure with contemporaneous recording of data can come both from studies using routinely recorded health data and primary data collection studies which directly recruit women during their pregnancies for longitudinal follow up during the postnatal years. Cohorts ascertained specifically for a specific research question offer the opportunity to collect data prospectively with methodological standardization of both procedures and measurements but can be challenged by poor or slow recruitment and high attrition levels (Charlton and de Vries, 2016). Attrition is a reality in longitudinal research when the observed outcomes are distal to the exposure event (pregnancy) but differ between primary and secondary data studies. For example, in the latter, attrition is generally lower and more a consequence of population movement between exposure types, movement between different healthcare providers who may not all provide data to the research dataset being utilized and via missing covariate values (e.g., nicotine use, family history of neurodevelopmental disorders). The NEWG gained consensus on the following statements relating to attrition:

- Attrition is an inherent aspect of longitudinal designs when neurodevelopmental measurements are administered directly to participants or where information on health and neurodevelopment is sought from the participant or health professional records over an extended period.
- The influence of attrition is a larger threat to the validity of the results when it is systematic and imbalanced across included groups, and this needs to be considered when interpreting study findings.
- Consensus on acceptable levels of attrition in longitudinal studies was difficult to achieve, and the need to adopt a pragmatic and realistic figure based on published and first-hand experience was recognized. Attrition rates of no greater than 20%–30% received support from 79% of the NEWG as being acceptable.
- Statistical methods investigating and addressing differences in demographics between the final study sample, and those lost to follow-up should be employed and reported.

Detailed information on the conduction of primary data cohort studies, in particular longitudinal cohort studies is beyond the scope of this paper, but readers are signposted to the important series of lessons learnt from environmental exposure research (Eskenazi et al., 2003; Dietrich et al., 2005). It was recognized by the NEWG that study designs using large epidemiological secondary health data have the strength of lower rates of attrition and large cohorts of representative samples of women as inherent aspects of their design.

#### 3.4.5 Comparator groups

**Table:** 

Recommendation 8
Comparator groups with different medication exposures for the same maternal disease(s) and groups with no medication exposure and no maternal disease exposure should be included in pregnancy pharmacovigilance study designs

Different comparator groups were viewed to be a critical aspect of PregPV studies for interpretation of collected data, to assist with balancing confounding or mediating factors and to facilitate understanding regarding the magnitude of the neurodevelopmental results.

There was strong consensus that the inclusion of both disease-matched treated and untreated groups was important to facilitate adjustment, at least in part, for any confounding due to the maternal disease for which treatment is indicated (“confounding by indication”), and for comparison to other available treatments. A co-recruited comparator group of pregnant women without the maternal disease and no exposure to a known teratogenic medication was also identified as important for situations where there is a potential for similar levels of risk from different treatments under investigation or for when there is potential risk associated with the maternal disease which needs to be quantified. Eighty-three percent of experts supported the inclusion of both an unexposed comparator group and specific medication comparator groups in PregPV studies for neurodevelopmental outcomes.

Fifty-eight percent of the NEWG supported a recommendation that comparator groups should be drawn from the same community as the exposure group(s) where possible to reduce ascertainment (sampling) bias. In certain situations, siblings with discordant medication exposures, children born to women who discontinued the investigated medication prior to pregnancy, or paternal exposure in a pregnancy where the mother is unexposed, where available, could provide an important comparator group option due to the likelihood of similar disease type and/or shared developmental environments, and as a means of balancing certain unmeasured confounding factors.

#### 3.4.6 Confounding and mediating variables

**Table:** 

Recommendation 9
The core set of influential variables, defined by the NEWG, should be included, and where required, adjusted for in pregnancy pharmacovigilance research investigating neurodevelopmental outcomes. Additionally, literature and expert input should determine whether there are any additional specific variables which should be included for the specific medication exposure and maternal disease indication under investigation

For a causal inference regarding a medication exposure and altered child neurodevelopmental trajectories to be valid, a range of co-occurring confounding, mediating and influential factors must be investigated through randomization, restriction, matching or other adjustment techniques. Given the range of environmental, familial and intrinsic influences on child neurodevelopment that occur in the general population and how these vary over the course of development, the NEWG set out to establish a list of important co-variables which should be considered across PregPV investigations regarding neurodevelopmental outcomes. In Round 1, an open question was used to elicit NEWG opinions on the variables they felt were important and in Round 2 this list was voted on in its entirety. A list of key variables with either confounding or mediator influences on neurodevelopmental outcomes across areas of the exposure, maternal history, and child factors was derived (Table 3).

**TABLE 3:** Mediating and confounding factors to be included in an optimal study design.

Domain	Agreement level
**Maternal factors**	
Indication	92.0%
SES	92.0%
Breastfeeding	89.5% (68.0%^a^)
Education	84.0%
Age	84.0%
IQ	80.0%
Caregiving environment	76.0%
Nutrition	72.0%
Parity	58.5% (56.0%^a^)
Marital Status	48.0%
**Other Exposures**	
Other prescribed medications	92.0%
Alcohol	92.0%
Other recreational drug use	88.0%
Tobacco	84.0%
**Paternal Factors**	
Education	80.0%
IQ	68.0%
**Medical & Family History**	
Neurodevelopmental Diagnoses or Difficulties	88.0%
Physical Illness	88.2% (76.0%^a^)
Mental Illness	92.0%
Genetic Disorder	92.0%
**Infant Factors**	
Prematurity	88.0%
Birthweight	84.0%
Sex	84.0%
Physical Illness	76.0%
Anthropometric Measures	68.0%
Early Intervention	68.0%

^a^ Round 2 voting before discussion and second voting in Round 3.

In addition to the list in Table 3, there may be other disease (including severity) or exposure specific variables which also require investigation, particularly if the maternal condition itself is also a risk factor for poorer child neurodevelopmental outcomes. Decisions regarding the potential influence of variables on child development should be made *a priori* and should be based on expert consensus and scientific understanding and should be measured reliably (Jacobson and Jacobson, 1996).

### 3.5 Improving reporting and interpretation of neurodevelopmental outcomes

**Table:** 

Recommendation 10
Reported results should be specific to the outcomes measured and the developmental period they were measured in. Reporting should include information on both the relative risk and absolute risk or effect size and include important contextual information

The complexity of brain functioning and its evolving capacity through to late adolescence/early adulthood can be a challenge for the reporting of outcomes. Further, the variety of approaches to measurement (e.g., psychometric tests which utilize continuous scales) or categorical classifications (e.g., diagnostic codes) can also make standardized reporting across an evidence base challenging. Considerations were given to this, and the following guidance was put forward by NEWG members:

- Study conclusions should be specific to the neurodevelopmental outcome investigated and the ages of the cohort, and over-generalization avoided.
- Studies should report both the relative and absolute risks and effect sizes and provide the context of the observed effect such as the national rates of a particular diagnosis or disorder or another clinical context.
- When measures have been used which employ a continuous measurement scale both the unstandardized effect size (e.g., loss of IQ points) and a standardized effect size (e.g., Cohen’s d (Lakens, 2013)) should be reported.
- Continuously measured traits often have cut off values which allow for dichotomization of a typical or atypical/poorer outcome. Whilst these provide an easier to communicate figure, it removes important information regarding the distribution of scores across the continuum (Cohen, 1983;Altman and Royston, 2006; Jacobson and Jacobson, 1996), including symptoms which sit subthreshold. It is recommended therefore that both continuous and dichotomous results are reported where available.

### 3.6 Barriers to undertaking research into neurodevelopmental outcomes

**Table:** 

Recommendation 11
Improved funding strategies for neurodevelopmental investigations are required which provide longer term infrastructure and expertise to support timely recognition of the potential later emergence of certain neurodevelopmental deficits

The NEWG members (84%) reported that there were significant barriers to neurodevelopmental research in PregPV. Sixty percent of the NEWG felt that there was a failure to prioritize neurodevelopmental outcomes within PregPV and that this was a barrier. However, challenges around the cost of neurodevelopmental investigations (92%) and funding such initiatives (84%) were by far the largest barriers identified. In Round 1, within-study design barriers such as recruitment, attrition and a lack of expertise were identified. However, 88% of experts felt that many of the in-study barriers could be overcome by using optimal study designs with improved funding. Over half of the NEWG felt that a lack of consensus regarding optimal study design (60%) and a lack of expertise internationally were also barriers.

Here we highlight that, despite the possible severity of impact on neurodevelopmental outcomes which place financial burdens on both families and society, the most common barrier to progress in obtaining appropriate evidence is funding. Optimal neurodevelopmental investigation requires adequate resources over a relatively long period, but the expense of these investigations should be weighed against the long-term cost to the individual and society where the neuroteratogenic effects of a medicine are undetected for considerable time periods. By reducing the time to detect signals, we stand to not only reduce the overall burden by reducing the number of exposures but also by providing early intervention to affected children to offset later emerging or cumulative impairments.

Current sources of funding for PregPV work comes from market authorization holders (pharmaceutical companies), public health bodies (select countries), government public health agencies and academic grant schemes. The delays in obtaining neurodevelopmental outcome data for specific medicines clearly illustrates the insufficiency of the current *ad hoc* funding model; a point made previously in relation to the antiseizure medications (Meador and Loring, 2016). Sustainable funding for PregPV studies was highlighted in an EMA public workshop (European Medicines Agency, 2020) and this NEWG adds to this call for improved mechanisms to fund neurodevelopmental outcome PregPV.

## 4 Discussion

In recent years, it has become apparent to academics, regulators, patients, pharmaceutical industry and healthcare professionals alike that the reproductive safety of medications cannot be assured without information about long-term neurodevelopmental outcomes. This consensus guideline provides 11 expert recommendations (Supplementary Table S4) regarding the integration and improvement of neurodevelopmental outcome investigations in routine PregPV initiatives. These recommendations are made with the desire to move forward to achieve robust evidence regarding neurodevelopmental risk more rapidly, thereby reducing the historical latency seen previously in medication safety processes (European Medicines Agency, 2020). Given the potential severity of the impact on brain development and functioning, there is an imperative to reduce the time taken to determine potential risk.

Randomized clinical trials to specifically investigate potential harms to fetal development are not possible, therefore observational trials are required. Given observational trials are susceptible to unmeasured confounding, complementary methodological approaches and replication is required. We realize that the recommendations made here set a high bar; however, no single study is necessarily expected to be able to be optimized in every one of these areas. Instead, these recommendations should be addressed across a complementary set of studies which converge to form a comprehensive set of evidence on which clinical and regulatory decisions can be made and advice to patients given. Over 90% of the NEWG supported the statement that a triangulation of evidence will be required from different sources and study designs to provide an optimal set of data on which to base conclusions upon. Data from preclinical/animal models, clinical and epidemiological studies are all required to inform each other and complement each other’s methodological strengths and weaknesses. Preclinical studies, allow tighter control over exposure factors (e.g., dose, duration, timing), the postnatal environment and behavioral experiments (Rice and Barone, 2000) and remove any potential influence from the maternal indication for treatment (e.g., disease or disorder). Prospective observational cohorts employing direct, blinded, and standardized psychometric assessments of included children, for example, offer unparalleled precision in measurement of the outcome and reduced bias due to standardization and blinding procedures. However, they are often at risk of selection biases and attrition. Routine use of healthcare data or educational data from large populations, on the other hand, allows for large and less selective populations to be investigated, does not require active participation from families and has less attrition.

The specific neurodevelopmental outcomes under investigation however may lend themselves more readily to specific data types or investigational approaches. Functional skills for example, such as intelligence or memory functioning are rarely measured routinely in healthcare settings but are frequently measured in the context of cohort studies. On the other hand, dichotomous outcomes, such as the presence or absence of a clinical diagnosis (e.g., ASD or ADHD) or examination grades, require a larger sample (Cohen, 1983), thus lending themselves towards secondary use of population level administrative records (Hjorth et al., 2019). Finally, whether the medicinal product is newly approved or already in widespread use will also be an important consideration in study design. Investigations into a newly approved medications, where there are a small number of exposed children initially, will be optimally investigated by designs with increased measurement sensitivity, which are accurate in the context of smaller group sizes and are able to detect early developmental deviations in infancy.

There are many medications currently in use which would be deemed important for investigation by the criteria in Recommendation 2. Whilst this poses practical and financial challenges for regulators and market authorization holders the importance of these outcomes and their potential life changing, and lifelong impacts mean that a multi stakeholder plan to address the knowledge gap for medications in widespread use is urgently required. An absence of evidence of fetal risk should not be confused as being evidence of fetal safety.

There was a strength of feeling in the NEWG that current funding models are a significant barrier to timely evidence generation. Regulators require companies with market authorization for the product to conduct or fund investigations, whilst clinical or academic based work is often subject to time limited and inconsistent funding streams; neither of which lead to trusted and timely evidence generation. Change is required to move forward with improving PregPV research with neurodevelopmental outcomes.

In summary, international medicines regulators should instruct post market authorization requirements which address each of the core neurodevelopmental outcomes. These investigations should be varied in their study designs to allow for the recommended triangulation of evidence, consider trajectories of skill maturation and the point at market authorization a medicine is at.

Our recommendations are the first regarding neurodevelopmental outcome PregPV investigations for medications and align with previous consensus initiatives regarding childhood vaccines and neonatal clinical trials with regards to early onset of investigations, longitudinal follow up, and standardized assessments (Marlow et al., 2019; Villagomez et al., 2019) and those for environmental neurotoxicology investigations, including the investigation of a range of neurodevelopmental domains, longitudinal design, confounding and mediator considerations, bias minimization and the emergence of effects later in the developmental trajectory (Amler et al., 2006). These recommendations should be utilized in conjunction with regulatory guidance (European Medicines Agency, 2019; U.S. Food and Drug Administration, 2019; Medicines and Healthcare Regulation Authority, 2021) on the undertaking of PregPV investigations to build evidence consensus for specific treatments.

Strengths of this work include its novelty, stakeholder engagement to determine information gaps and a rigorous Delphi approach to develop consensus. Included experts were from environmental and substances of abuse neurotoxicology research groups as well as PregPV researchers. Both academic and clinical experts were from different countries and were experienced in different research designs and data sources. We acknowledge however that these guidelines however are limited in certain areas. Firstly, opinions were ascertained on optimal features of investigations which were not bound by feasibility constraints, different PregPV stakeholders are encouraged to review how these recommendations can inform their future work. Secondly, the incorporation of culturally and linguistically sensitive measurements is a further important consideration when designing studies across different countries and cultures (Villagomez et al., 2019) but was not covered here. Thirdly, the NEWG were not able to produce guidance on a range of additional outstanding issues including harmonization of data collection, measurement choice/standardization or optimal statistical analysis approaches. It is recognized that these are required and should be addressed in future expert consensus work. Finally, we were not able to take these 11 recommendations back to the original stakeholders or to collect wider endorsement prior to publication. However, it is the intention that these recommendations are the genesis of a wider conversation over the improvement of PregPV research where there are neurodevelopmental outcomes.

## 5 Conclusion

The reproductive safety of medicines includes knowledge on long-term neurodevelopmental outcomes. A set of core neurodevelopmental outcomes are proposed, which require *a* triangulation of evidence from different study designs and data sources. We put forward 11 recommendations to improve neurodevelopmental investigations which will reduce the risk to the fetus and increase maternal confidence in medication use during the childbearing years. Successful and timely understanding of neurodevelopmental risk from fetal exposure to medications will require improved funding for both pre-clinical and human investigations to delineate these risks.

## Data Availability

The original contributions presented in the study are included in the article/Supplementary Material, further inquiries can be directed to the corresponding author.
