# Supplementary material for: Expert consensus on neurodevelopmental outcomes in pregnancy pharmacovigilance studies
Source: Front Pharmacol. 2023 Jun 1;14:1094698. doi: 10.3389/fphar.2023.1094698 (PMC10270323; doi:10.3389/fphar.2023.1094698)
Supplement: Supplementary file 1 [file Table4.pdf]

Supplementary Table 4 | Recommendations for investigation of Neurodevelopmental outcomes in PregPV investigations

1.	Neurodevelopmental outcomes should be integral to pregnancy pharmacovigilance.
2.	Neurodevelopmental investigations should automatically be part of the PregPV initiatives for a medication where one or more apply:<br>i) It is mechanistically plausible for the medication to be associated with an increased risk or it belongs to a class of medications where effects have been observed (e.g., central nervous system acting medications).<br>ii) There is evidence (preclinical or human data) of a higher risk to physical development (e.g., structural anomalies),<br>iii) There is evidence (preclinical or human data) of a higher risk to brain development<br>iv) The medication is likely to be widely used among women of childbearing potential.
3.	An evidence base must include assessment of these expert agreed neurodevelopmental functions. These include cognitive, motor, behavior and emotional functioning as well as clinical disorders and educational outcomes.
4.	Neurodevelopmental outcomes are diverse and require different measurement approaches. Use of a standardized set of direct assessments, by trained assessors blinded to exposure status for the whole cohort receives the highest recommendation in terms of measurement sensitivity.
5.	Factors related to the medication exposure including the dose, gestational timing, duration, and route of administration during pregnancy should be key considerations and study designs should take heterogeneity in these factors into account.
6.	The investigation of neurodevelopmental outcomes should start in infancy with an extended period of investigation into adolescence. Investigation or sampling intervals should be more frequent during periods of rapid development or after signals of early developmental deviations.
7.	Research can be improved by using a prospective design, representative samples of women using the medication and through limited attrition which is not systematic and imbalanced across groups.
8.	Comparator groups with different medication exposures for the same maternal disease(s) and groups with no medication exposure and no maternal disease exposure should be included in pregnancy pharmacovigilance study designs.
9.	The core set of confounding variables, defined by the NEWG, should be included, and where required, adjusted for in pregnancy pharmacovigilance research investigating neurodevelopmental outcomes. Additionally, literature and expert input should determine whether there are any additional specific variables which should be included for the specific medication exposure and maternal disease indication under investigation.
10.	Reported results should be specific to the outcomes measured and the developmental period they were measured in. Reporting should include information on both the relative risk and absolute risk or effect size and include important contextual information.
11.	Improved funding strategies for neurodevelopmental investigations are required which provide longer term infrastructure and expertise to support timely recognition of the potential later emergence of certain neurodevelopmental deficits.
