# Supplementary material for: Expert consensus on neurodevelopmental outcomes in pregnancy pharmacovigilance studies
Source: Front Pharmacol. 2023 Jun 1;14:1094698. doi: 10.3389/fphar.2023.1094698 (PMC10270323; doi:10.3389/fphar.2023.1094698)
Supplement: Supplementary file 2 [file Table1.pdf]

Supplementary Table 1| Examples provided for the five identified themes.

Theme	Subthemes
Importance and timing of investigations	• Are neurodevelopmental outcomes important enough to be considered central to pregnancy pharmacovigilance or collected routinely?<br>• Which products should neurodevelopmental outcomes be investigated for?<br>• What data should prompt neurodevelopmental investigations?
Core Outcomes	• What aspects of neurodevelopment are considered key/ core outcomes?
Optimal Methodology	• What is the optimal approach to investigation?<br>• What are the key confounder and medicator variables which should be considered?
Age of Investigation	• What is the optimal time to assess neurodevelopmental outcomes?
When is Evidence Conclusive?	• How should data be interpreted?<br>• When can conclusions be firmly drawn?
