# Supplementary material for: Expert consensus on neurodevelopmental outcomes in pregnancy pharmacovigilance studies
Source: Front Pharmacol. 2023 Jun 1;14:1094698. doi: 10.3389/fphar.2023.1094698 (PMC10270323; doi:10.3389/fphar.2023.1094698)
Supplement: Supplementary file 3 [file Table2.pdf]

Supplementary Table 2 | Round 1 Questions to the NEWG

Theme	Question
**At what point following approval should human neurodevelopmental outcomes be assessed?**	• Should child neurodevelopment be investigated routinely for all medications following approval for use in humans?<br>• Are there certain medication exposures which should be prioritised for neurodevelopmental investigation?
**Barriers to neurodevelopmental outcome research**	• Are pre-clinical (animal) study results predictive of human neurodevelopmental outcomes<br>• Are there any barriers to researching neurodevelopmental outcomes in children exposed in the womb?
**Core Outcomes**	• Based on your informed professional opinion, which aspects of neurodevelopment can be altered by prenatal exposures (chemical, environmental or medicinal)?<br>• Can a single score or single aspect of neurodevelopment be used to summarize neurodevelopmental functioning in all other areas?<br>• What areas of neurodevelopment should be investigated before any conclusion can be made regarding risk or safety for a specific medication (e.g. by regulators, prescribers)?<br>• What type(s) of investigations into neurodevelopment should be completed before conclusions can be made regarding either risk or safety for a specific medication?
**Optimisation of Measurement**	• What is the optimal way to measure neurodevelopment in a group of children exposed in utero?<br>• At what age should the investigation of neurodevelopmental outcomes start?<br>• At what age should the investigation of neurodevelopmental outcomes in exposed cohorts end?<br>• At what age interval should longitudinal follow-up studies assess neurodevelopment?<br>• Should the interval of assessment be different for exposures with early deviations in developmental trajectories versus those where development was similar to control children in infancy?<br>• In your informed professional opinion, what variables are important to adjust for when investigating the impact of a given exposure?<br>• In your informed professional opinion, what details about the exposure are important to record and consider?<br>• We want to understand the optimal design features for the investigation of an exposure on child neurodevelopment. Please describe the ways that the methodology could be optimized in the following areas: Participant selection, Control/comparator, Exposure data, Measurement, Confounding optimization, Attrition, Other important design.
**Interpretation**	• What is good practice in the interpretations and reporting of outcomes?<br>• How do we best convey the results from measures (e.g. questionnaires, psychometrics) which score on a continuous measurement?<br>• How would you best communicate mean differences in infant/child outcomes in a way that is meaningful?
**Other**	• Is there any other topic in relation to neurodevelopmental outcomes which you would like to include in these discussions?
