# Supplementary material for: Expert consensus on neurodevelopmental outcomes in pregnancy pharmacovigilance studies
Source: Front Pharmacol. 2023 Jun 1;14:1094698. doi: 10.3389/fphar.2023.1094698 (PMC10270323; doi:10.3389/fphar.2023.1094698)
Supplement: Supplementary file 4 [file Table3.pdf]

Supplementary Table 3| Round 2 Questions to the NEWG

Topic/Rating Instructions	Statements
**Routine Assessment**<br><br>Please rate the extent to which you agree or disagree with the importance of each of the following factors in selecting particular medications for routine human research:	• Central Nervous System acting medications.<br>• Medications with documented impact on the physical development of the fetus (i.e. malformation, dysmorphic facial features, growth restriction).<br>• Evidence of risk from animal studies.<br>• Medications that are widely used in female populations and thus the rate of fetal exposure is high.<br>• Is related either by therapeutic class or mechanism of action to a medication known already to have impact on the development of the human brain<br>• Published case reports, pilot studies or other early evidence indicating potential risk or association with impairment.<br>• A signal or risk generated through industry or regulatory adverse event reporting systems.
**Pre-Clinical Studies**	• Where animal studies indicate risk for a specific medication this medication should automatically be subject to routine follow-up in humans.<br>• Where animal studies do NOT indicate risk for a specific medication this cannot be interpreted as evidence of safety in humans.
**Barriers to Research**<br><br>Please indicate the extent to which you disagree or agree that each of the following represents a barrier to research in this area:<br><br>Please rate your level of agreement with the following statement:	• Difficulty obtaining funding is a barrier.<br>• A view that neurodevelopmental functioning is less important or of less significance than physical child outcomes is a barrier.<br>• Cost of follow-up to investigate long-term effects is a barrier<br>• An international lack of expertise required for assessment and interpretation of neurodevelopmental outcomes is a barrier<br>• Lack of consensus regarding approach to study design is a barrier<br>• In an ideal world, in which funding is ample, many of the barriers to child neurodevelopmental research can be overcome by using optimal study design
**Single vs Multiple Scores/Domains**	• To what extent do you agree that no single score or domain can reliably inform on the functioning of all other neurodevelopmental domains?
**Core Outcomes**<br><br>Please indicate your level of agreement or disagreement that each of the domains listed below can be altered by prenatal exposure.	• Cognitive<br>o General cognitive skills (e.g. developmental quotient, intelligence quotient, IQ); Expressive Language; Receptive Language; Executive Function; Learning rate; Memory; Attention; Processing; Visuo-Spatial skills<br>• Social/Behavioural<br>o Adaptive behaviour (e.g. daily living skills); Behavior Problems; Social skills<br>• Motor<br>o Developmental milestones; Reflexes; Fine motor; Gross motor<br>• Emotion and mood difficulties<br>o Emotional regulation difficulties; Anxiety; Depression<br>• 'Real world' outcomes<br>o Examination results; Rates of specialist educational needs/ support; Occupational attainment<br>• Clinical Disorders
**Core Domains**	• Cognitive

Please indicate what you think should be included in a **CORE** or **CENTRAL** set of neurodevelopmental outcomes that would require investigation across studies before conclusions can be made regarding the potential longer term impact on child neurodevelopment.	• Adaptive behaviour (e.g. daily living skills)<br>• Social skills<br>• Motor<br>• Behavior Problems<br>• Emotion and mood difficulties<br>• 'Real world' outcomes<br>• Clinical Disorders<br>• Sexual Function
**Optimal Study Design**<br><br>Please indicate your level of agreement or disagreement with the following statement:	• A variety of investigative approaches including prospective, longitudinal studies, routine healthcare records, and animal data is required to ensure optimisation of data collection in this area.
**Optimal Design Features**<br><br>Please indicate your agreement or disagreement with the following statements regarding an optimal approach to outcome measurement:	• The optimal research strategy for neurodevelopmental domains would involve direct assessment of the child for the purpose of the study?<br>• For some neurodevelopmental skills or domains such as Cognitive or Motor functioning researcher administered assessments would be optimum, whilst for others domains parental completed questionnaires may be more applicable (e.g. behaviour, social skills, mood).<br>• Measurements of the different aspects of neurodevelopment (whether researcher or parent completed) should be undertaken using standardised and validated measures?<br>• Assessments should be administered by experts in the relevant field.
**Optimal Timing**<br><br>Please indicate your level of agreement or disagreement with the following statements:	• The optimal research design for investigations of neurodevelopmental outcomes would involve long-term follow-up that begins in early infancy and continues into early adulthood.<br>• Many neurodevelopmental impairments may only become fully apparent as the child gets older.<br>• Assessments in infancy (=/<24 months) are useful for identifying early deviations in neurodevelopmental development.<br>• Assessments in school-age children (4-5+ years) provide more useful and reliable information than assessments carried out during infancy (0-2 years).<br>• Assessments in the early adulthood would provide the most reliable information regarding functioning as all neurodevelopmental domains are reaching full maturity.
Please indicate what you think is the ideal starting point for neurodevelopmental assessments following exposure to medication in the womb:	• In utero<br>• 6 months<br>• 2- years<br>• 4-5 years
Please indicate what you think is the ideal (funding no issue) end-point for neurodevelopmental assessments following exposure to medication in the womb:	• 4-5 years<br>• 8-10 years<br>• 12 years<br>• 16 years<br>• 21 years<br>• 30 years
Can final conclusions regarding the level of risk a medication is associated with be drawn following assessments in infancy only?	• Yes<br>• No
**Optimal Intervals**	• It is important to develop an assessment protocol across different stages of development in order to facilitate the building of a

Please indicate your agreement or disagreement with the following statements:	comprehensive understanding of the developmental trajectory of core neurodevelopmental outcomes<br>• Assessments should be more frequent during infancy and decrease as the child gets older.<br>• Development occurs at a much higher rate during infancy and so requires more regular assessments.<br>• Development occurs at a slower rate once the child reaches school age and so less regular assessments are required.
Please indicate your agreement or disagreement with the following statements:	• Early deviation in neurodevelopmental trajectories following exposure to medication in utero suggest that more intensive follow-up is required.<br>• Lack of early deviation in neurodevelopmental trajectories does not mean later effects will not emerge, and so regular follow-up is still required.
**Confounding**<br><br>Tick all that you feel should be included in an optimal study design when the primary outcome is a neurodevelopmental domain:	• Maternal Factors<br>o Indication; IQ; Education; Age; Parity; Marital Status; Socio-economic status; Nutrition; Breastfeeding; Caregiving environment<br>• Paternal Factors<br>o IQ; Education<br>• Other Exposures<br>o Other prescribed medications; Alcohol; Tobacco; Other recreational drug use.<br>• Medical and Family History<br>o Neurodevelopmental problems; Physical illness; Mental illness; Genetic disorder<br>• Infant Factors<br>o Birthweight; Anthropometric data (length, head circumference etc); Sex; Prematurity; Physical illness; Physical injury; Early intervention
**Exposure Details**<br><br>There was a clear theme in the comments that dose, timing and duration of the exposure were important considerations. To help us formalise this level of agreement to what extent do you agree that the following information should be considered in any analysis:	• Dose of exposure<br>• Timing of exposure during pregnancy (e.g. onset in 1st, 2nd, 3rd trimester etc.)<br>• Duration of exposure<br>• Changes to medication during pregnancy/continuous measure of exposure<br>• Route or mechanism of the drug<br>• Patient compliance/adherence to prescribed dose<br>• Continued collection of data throughout breastfeeding.<br>• Exposure to maternal condition (e.g. seizures).<br>• Biomarkers of the level of the medication present in the infant.<br>• Co-medications
**Recruitment (Case):**<br><br>To what extent to you agree that each of these statements would provide an optimal participant selection:	• Prospective recruitment of participants during pregnancy for longer term follow up<br>• A sample that is representative of the relevant population (e.g. large sample, or through population-wide registers, multi-centre recruitment etc)<br>• Pre-defined and standardised inclusion and exclusion criteria<br>• Recruitment of participants for neurodevelopmental follow up from existing prospective medical or pregnancy registers (e.g. disease specific registers such as a register for women with epilepsy where initial recruitment was only focused on immediate birth outcomes)

**Recruitment (Control/comparator)**<br><br>To what extent do you agree that the following provide one of the needed control/ comparator groups for medication exposure studies:	• The recruitment of participants who have been exposed to the same disease but a different medication<br>• The recruitment of participants who have been exposed to the same disease but have not received prescribed medication.<br>• The recruitment of participants who have not been exposed to the disease.<br>• Do you agree that studies should recruit both comparator (e.g. same disease with different drug or no drug) AND control (e.g. no disease or drug) groups?<br>• Control/comparators should be matched to exposed group on key maternal variables.<br>• Control comparators should be recruited from the same clinic/community as the exposed group.
**Exposure Data Ascertainment**<br><br>A number of methods for ascertaining exposure data were suggested by respondents. Please rank these in order of reliability, with 1 being the most reliable and x being acceptable but with limitations:	• Prospective concurrent self-report of medication use by women themselves<br>• Collection of biomarker data (e.g. serum levels, umbilical cord blood)<br>• Collection of medication exposure data from medical records<br>• Collection of medication exposure data from pharmacy dispensing records.<br>• A combined approach of collecting both self-report data from the mother and supplementary secondary data from medical records/ pharmacy dispensing records
**Measurement of Neurodevelopment**<br><br>To what extent do you agree with the following statements regarding the optimal approach to outcome data collection:	• For certain outcome (e.g. infant development or child cognitive functioning) direct researcher led assessments are optimal (gold standard)<br>• For other outcomes (e.g. child behaviour or social development) parent completed questionnaires are optimal<br>• Assessments should be standardised across all included children<br>• The assessment should be blinded to the exposure history of the child<br>• Assessments should be administered by a small pool of researchers to reduce issues of inter-rater reliability.
**Confounding Optimization**<br><br>Do you agree that the optimal way of selecting confounders for specific studies involves the following	• The development of a core set of pre-defined factors that have been discussed and agreed upon by experts in the field.<br>• Although it may be possible to develop a core set of confounders, it remains important to consider and include additional confounders that may be of specific relevance to a given medication.<br>• Any decisions regarding confounder or mediator selection should be made *a priori* and based on previous literature or scientific knowledge<br>• Adequate consideration should be given to the distinction between *confounders* and *mediators,* to avoid masking potential and important indirect effects.<br>• The use of novel techniques to detect previously unmeasured confounding variables (e.g. natural experiments, machine learning etc.)<br>• Where the siblings are discordant for the medication exposure in question this addresses a number of important confounders
**Attrition**	• Attrition is an inherent characteristic within longitudinal cohort designs

Please indicate the extent to which you agree or disagree with the following statements:	• Systematic attrition (where one group or factor is more inclined to be lost to follow up) creates more of a challenge to the reliability of the results than a balanced attrition<br>• What is an acceptable level of attrition in longitudinal studies, following patients for a number of years:<br>o 10%<br>o 20%<br>o 30%<br>o 40%<br>• Predetermined statistical methods for dealing with unbalanced attrition should be in place.
**Good reporting**<br><br>Please indicate the degree to which you agree or disagree with the following statements regarding good practice in reporting and interpreting results	• Absolute risk statistics and effect sizes (e.g. percentages, numbers within clinical ranges, mean differences etc) as well as relative risk or odds ratios results should be reported.<br>• The context of the size of the effect or difference should be described with age related and national norms.<br>• Standardised reporting of effect sizes Cohen’s d would assist in comparing outcomes across studies and measures<br>• Authors should provide context on how any effect might impact an individual’s educational, occupational and relational attainment (where relevant).
